# Neutralization of CD95 ligand protects the liver against ischemia-reperfusion injury and prevents acute liver failure

**DOI:** 10.1038/s41419-017-0150-0

**Published:** 2018-01-26

**Authors:** Mohammed Al-Saeedi, Niels Steinebrunner, Hassan Kudsi, Niels Halama, Carolin Mogler, Markus W. Büchler, Peter H. Krammer, Peter Schemmer, Martina Müller

**Affiliations:** 1 Department of General, Visceral, and Transplant Surgery, Heidelberg University Hospital, Heidelberg, Germany; 2 Department of Gastroenterology, Intoxications, and Infectious Diseases, Heidelberg University Hospital, Heidelberg, Germany; 3 Medical Oncology, National Center for Tumor Diseases, University of Heidelberg, Heidelberg, Germany; 4 Department of Pathology, Heidelberg University Hospital, Heidelberg, Germany; 5 Division of Immunogenetics, German Cancer Research Center, Heidelberg, Germany; 6 Department of Surgery, Division of Transplant Surgery, Medical University of Graz, Graz, Austria; 7 Department of Internal Medicine I, Gastroenterology, Endocrinology, Rheumatology, and Infectious Diseases, Regensburg University Hospital, Regensburg, Germany

## Abstract

Ischemia-reperfusion injury is a common pathological process in liver surgery and transplantation, and has considerable impact on the patient outcome and survival. Death receptors are important mediators of ischemia-reperfusion injury, notably the signaling pathways of the death receptor CD95 (Apo-1/Fas) and its corresponding ligand CD95L. This study investigates, for the first time, whether the inhibition of CD95L protects the liver against ischemia-reperfusion injury. Warm ischemia was induced in the median and left liver lobes of C57BL/6 mice for 45 min. CD95Fc, a specific inhibitor of CD95L, was applied prior to ischemia. Hepatic injury was assessed via consecutive measurements of liver serum enzymes, histopathological assessment of apoptosis and necrosis and caspase assays at 3, 6, 12, 18 and 24 h after reperfusion. Serum levels of liver enzymes, as well as characteristic histopathological changes and caspase assays indicated pronounced features of apoptotic and necrotic liver damage 12 and 24 h after ischemia-reperfusion injury. Animals treated with the CD95L-blocker CD95Fc, exhibited a significant reduction in the level of serum liver enzymes and showed both decreased histopathological signs of parenchymal damage and decreased caspase activation. This study demonstrates that inhibition of CD95L with the CD95L-blocker CD95Fc, is effective in protecting mice from liver failure due to ischemia-reperfusion injury of the liver. CD95Fc could therefore emerge as a new pharmacological therapy for liver resection, transplantation surgery and acute liver failure.

## Introduction

Hepatic ischemia-reperfusion injury (IRI) is a pathophysiological process in liver surgery and transplantation, as well as in trauma, hypovolemic shock and sepsis. In an effort to ameliorate the problem of the severe shortage of available organs for transplantation, grafts from extended criteria donors, which are often highly susceptible to IRI, are increasingly being used for transplantation. Thus, there is an urgent clinical need for protective strategies against IRI in order to promote better graft function^1–5^.

IRI of the liver is mediated by a complex series of mechanisms, including the production of reactive oxygen species (ROS), as well as local and systemic inflammatory responses, which are mediated by the release of pro-inflammatory cytokines from innate immune cells (i.e. Kupffer cells). Furthermore, activated macrophages, cytotoxic T lymphocytes and natural killer (NK) cells tend to migrate to the liver^6^, resulting in the death of endothelial lining cells and hepatocytes via a multistep process involving both apoptosis and necrosis^7–10^. Given the prolific expression of death receptors on hepatocytes, primarily of the death receptor CD95 (Apo-1/Fas), the liver is particularly susceptible to death receptor-mediated apoptosis^7,11–14^. Upon activation by the CD95 ligand (CD95L), the oligomerization of the CD95 death receptor leads to the recruitment of cytoplasmic adaptor proteins, which activate apical caspases of the apoptotic signaling pathway, mainly caspase-8 and caspase-9. These subsequently mediate the recruitment of downstream effector caspases, such as caspase-3, in the execution phase of apoptosis^15–20^.

Using a mouse nonlethal hepatic ischemia-reperfusion (IR) model, we assessed the role of the CD95 signaling pathway for the development of IRI. Based on the assumption that the CD95 death-receptor system might essentially determine IR-related liver damage, we set up a novel intervention strategy with a CD95Fc decoy construct consisting of the extracellular domain of the CD95 receptor and the Fc domain of an Immunoglobulin G (IgG) antibody^21^. CD95Fc binds to the CD95L, thereby inhibiting the activation of the CD95 pathway by CD95L. CD95Fc-mediated blocking of the CD95 signaling pathway significantly attenuated CD95-mediated apoptosis and toxicity following IRI. Inhibition of the CD95 signaling pathway may thus represent a novel and promising approach to protect the liver against IRI-related diseases.

## Materials and methods

### Reagents

The CD95Fc decoy construct, herein referred to as CD95Fc, consists of the extracellular domain of the CD95 receptor and the Fc domain of an IgG antibody. CD95Fc binds to the CD95L, thereby inhibiting the activation of the CD95 pathway by CD95L. CD95Fc was kindly provided by Apogenix GmbH (Heidelberg, Germany). IgG was purchased from Talecris Biotherapeutics (Frankfurt, Germany).

### Animal model

Ten-week-old male C57BL/6 mice (Charles River Laboratories, Sulzfeld, Germany) were used in all experiments. The animals received humane care, had free access to food and water, and were kept on a 12-h light/dark cycle in a temperature-controlled room. The Animal Care and Use Committee of the University of Heidelberg approved the protocol.

Normothermic ischemia was applied to the liver of the animals followed by reperfusion, as described previously^22,23^. The animals were anesthetized briefly with xylazine 10 mg/kg and ketamine 100 mg/kg by intraperitoneal injection. The animals’ body temperature was maintained with a warming pad. After median laparotomy, the liver was mobilized. The median and left liver lobes, which together make up about 70% of the liver mass, were clamped with an atraumatic microvascular clamp (Fine Science Tools, Heidelberg, Germany) at its base, including all structures of the portal triad (the hepatic artery, the portal vein, and the bile duct). Using this method, an external shunt can be avoided because the blood flow is directed through the right and caudate lobes, thereby preventing mesenteric venous congestion^22,24^. Reperfusion was initiated by removal of the clamp after 45 min. Sham-operated animals underwent identical anesthetic and surgical procedures, but had no clamp application.

The animals in the treatment group received a total of two intravenous (IV) injections of CD95Fc (30 mg/kg) at 12 h and at 30 min before warm ischemia. Control animals received an analogous dosage of IgG (30 mg/kg). The animals were sacrificed at 3, 6, 12, 18 and 24 post ischemic hours and blood and tissue samples were harvested. Serum levels of alanine aminotransferase (ALT), aspartate aminotransferase  (AST), and lactate dehydrogenase (LDH) increased progressively after the 45 min ischemic insult and peaked at 12 h after reperfusion. Thereafter, serum levels decreased at 18 and 24 post ischemic hours in accordance with enzymatic half-life variations. We observed a significant decrease of the levels of ALT, AST and LDH in CD95Fc-treated animals in comparison with IgG-treated animals for the time points of 6, 12, 18, and 24 post ischemic hours (Fig. 2). The time points of 12 and 24 h of reperfusion were chosen for further serological and histopathological analyses.

**Fig. 1:**
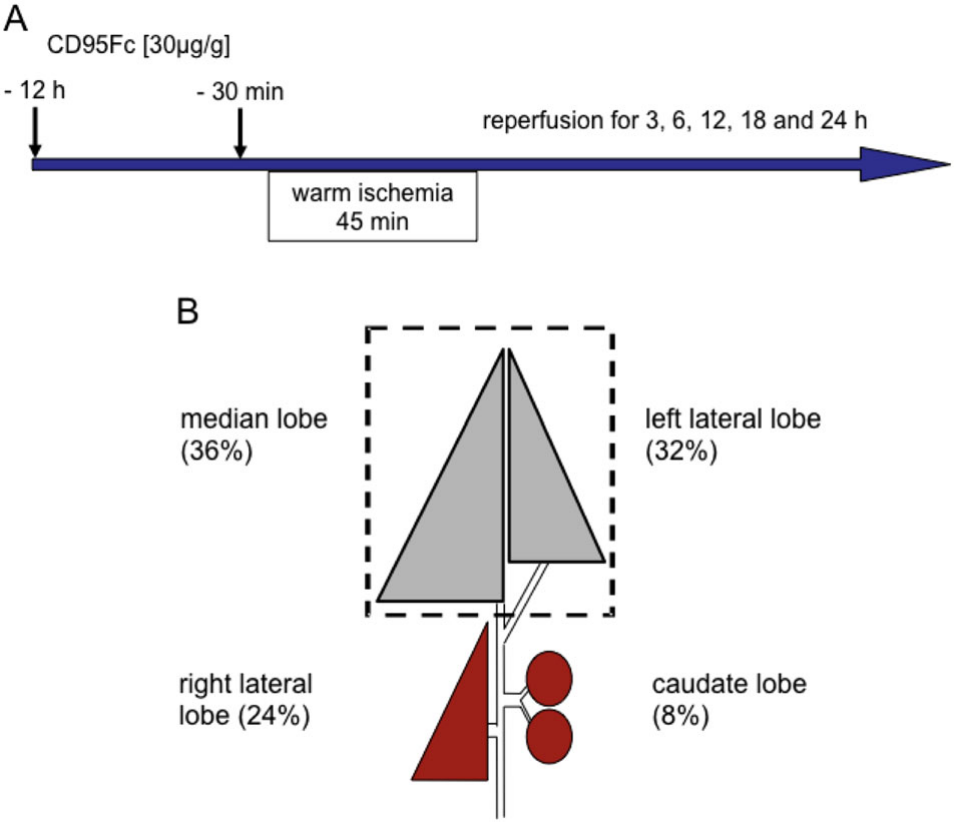
Experimental design **a** and **b** Animals received a total of two IV injections of CD95Fc (30 mg/kg) at 12 h and again at 30 min before the induction of 45 min of warm ischemia of the median and left lateral liver lobe (68% of the liver). Control mice received the same volume of IgG (30 mg/kg) antibody. Mice were sacrificed after 3, 6, 12, 18 and 24 h following reperfusion and blood and tissue samples were harvested for serological and histological assessment of liver damage.

Whole blood samples were allowed to clot and then centrifuged at 1000×*g* for 5 min. Serum was collected and stored at −80 °C. Liver sections were either fixed in 4% phosphate-buffered formalin and subsequently embedded in paraffin or snap frozen in liquid nitrogen and stored at −80 °C for histological analysis. The experimental design is outlined in Fig. 1a, b.

### Assays

Serum ALT, AST and LDH were measured in the Institute of Clinical and Laboratory Medicine at the University Hospital Heidelberg according to standard procedures.

For detection of caspase activity, caspase-3, caspase-8 and caspase-9 fluorometric assays (R&D Systems, Wiesbaden, Germany) of protein extracts of homogenated tissue from post ischemic livers were performed, as previously described^25^.

### Histology

For terminal deoxynucleotidyl transferase-mediated dUTP nick-end labeling (TUNEL) staining, cryosections of the mouse livers (5 µm in thickness) were stained using the In Situ Cell Death Detection Kit (Roche Diagnostics, Indianapolis, IN, USA), as described in the manufacturer’s instructions. For subsequent evaluation, slides were scanned using a NDP NanoZoomer (Hamamatsu Photonics, Japan). The VisioMorph software system (Visiopharm, Denmark) was used for automated image analysis (i.e. staining intensity analysis, quantification, etc., as previously described^26–28^. A specific protocol was developed to enable the software to distinguish between TUNEL-positive and TUNEL-negative cells. TUNEL-negative staining on three heterogenic, full slide sections (tissue artifacts were excluded from analysis by visual inspection), and TUNEL-positive staining were defined by two observers (N.S. and N.H.). Three independent full slide sections were then used to validate the approach.

After validation, tissue samples from all mice (three full sections from each mouse and serial sections with spacing of 30 µm) were included in the analysis. As reproducibility of corresponding sections from the same mice was excellent (Spearman’s rank correlation, *r* = 0.92, *p* = 0.001), data analyses for all three sections from each mouse were pooled. For each section, the percentage of TUNEL-positive tissue surface area was calculated as follows:

$$\left[ {{\rm {TUNEL}}-{\rm{positive}}\,{\rm{tissue}}\,{\rm{surface}}\,{\rm{area}}/\left( {{\rm{complete}}\,{\rm{section}}\,{\rm{area}} - {\rm{background}}\,{\rm{area}} - {\rm{tissue}}\,{\rm{artifacts}}} \right)} \right]^\ast 100$$

For evaluation of necrosis, the livers were fixed in 4% buffered formalin and embedded in paraffin. Sections (3 µm in thickness) were cut and H&E staining was performed according to standard protocols. Slides were evaluated without knowledge of the origin of the specimens and with special regard to liver architecture, cellular changes, and extent of necrosis (% of liver).

### Statistical analysis

Variables are expressed as mean values and standard deviation (S.D.). We applied multivariate analysis of variance (MANOVA) to test for statistical significance. Statistical analysis was carried out using the SAS software system (SAS Institute Inc., Cary, NC, USA). A *p*-value of less than 0.05 was considered statistically significant.

## Results

### CD95Fc decreases liver enzymes after hepatic IRI

**Fig. 2:**
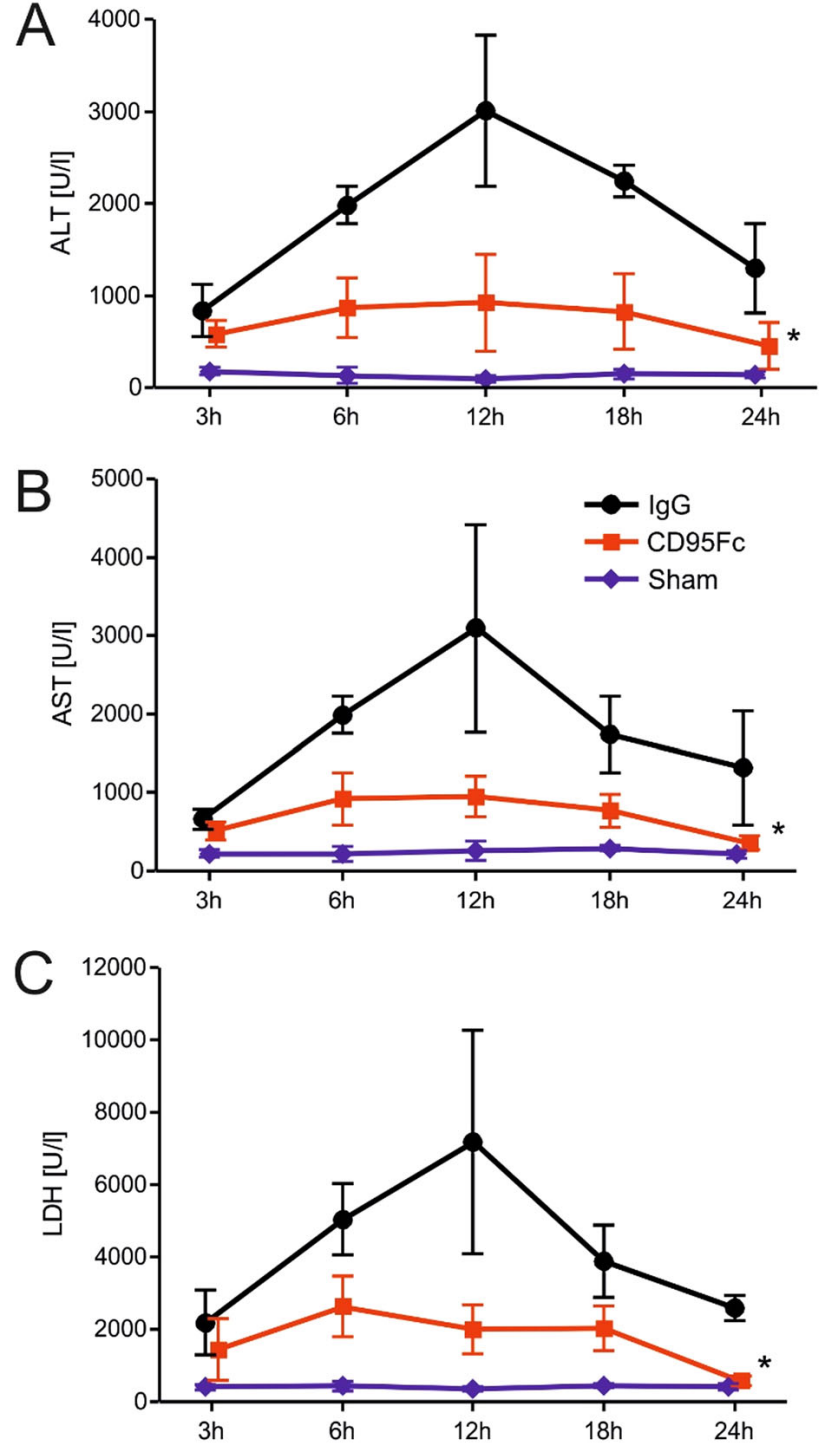
Serum liver enzymes (transaminases and lactate dehydrogenase) are reduced by application of CD95Fc Following 45 min of warm ischemia of the median and left lateral liver lobe, serum was collected after 3, 6, 12, 18 and 24 h from CD95Fc-treated and control mice. ALT alanine aminotransferase **a**, AST aspartate aminotransferase **b**, and LDH lactate dehydrogenase **c** were measured (mean ± S.D., *n* = 5. *p < 0.0001, MANOVA, between-subject-effect compared to IgG, time points 3, 6, 12, 18 and 24 h were considered as repeated measurements)

Using a mouse nonlethal hepatic IRI model, we found that neutralization of CD95L significantly decreased IR-induced liver damage assessed by elevated liver enzymes and LDH. The levels of ALT, AST, and LDH in control mice reached values of 2727 ± 1002 U/l, 2713 ± 1505 U/l and 6550 ± 3166 U/l, respectively, whereas the presence of CD95Fc significantly reduced hepatic injury by 35% for ALT (*p* = 0.0014), 40% for AST (*p* = 0.0074), and 43% for LDH (*p* = 0.0064) 12 h after reperfusion in the treated animals. This effect was still present 24 h after reperfusion. ALT, AST and LDH values were lowered by 46% (*p* = 0.0089), 34% (*p* = 0.0201) and 30% (*p* < 0.0001), respectively, in the CD95Fc-treated animals vs. the control animals (Fig. 2a–c).

Thus, neutralization of CD95L significantly ameliorates hepatocyte damage of mouse livers which have undergone IRI.

### Neutralization of CD95L exerts hepatoprotective effects

**Fig. 3:**
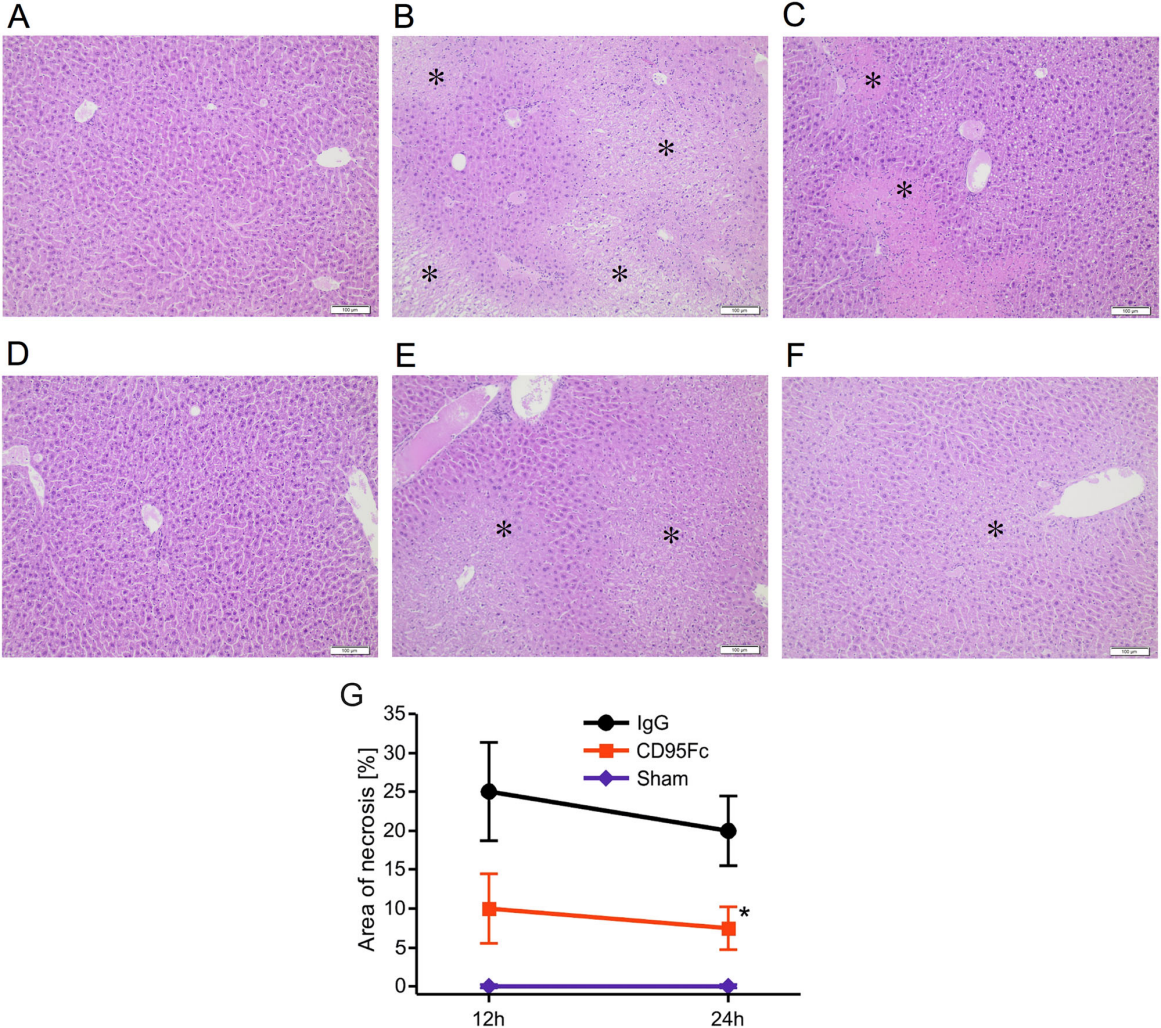
Hepatocellular necrosis is ameliorated by the application of CD95Fc Liver injury was induced by 45 min of warm ischemia of the median and left lateral liver lobe after prior application of two IV injections of CD95Fc (30 mg/kg) or the same volume of IgG (30 mg/kg) antibody at 12 h and at 30 min before ischemia. After 12 h and again after 24 h of reperfusion, liver tissue was harvested and processed for histopathology. Representative H&E-stained liver sections (×100 magnification, areas of necrosis marked with an asterisk). **a** sham, reperfusion 12 h; **b** IgG, reperfusion 12 h; **c** CD95Fc, reperfusion 12 h; **d** sham, reperfusion 24 h; **e** IgG, reperfusion 24 h; **f** CD95Fc, reperfusion 24 h. **g** Area of necrosis (% of area) (mean ± S.D., *n* = 6. *p < 0.0001, MANOVA, between-subject-effect compared to IgG, time points 12 and 24 h were considered as repeated measurements)

**Fig. 4:**
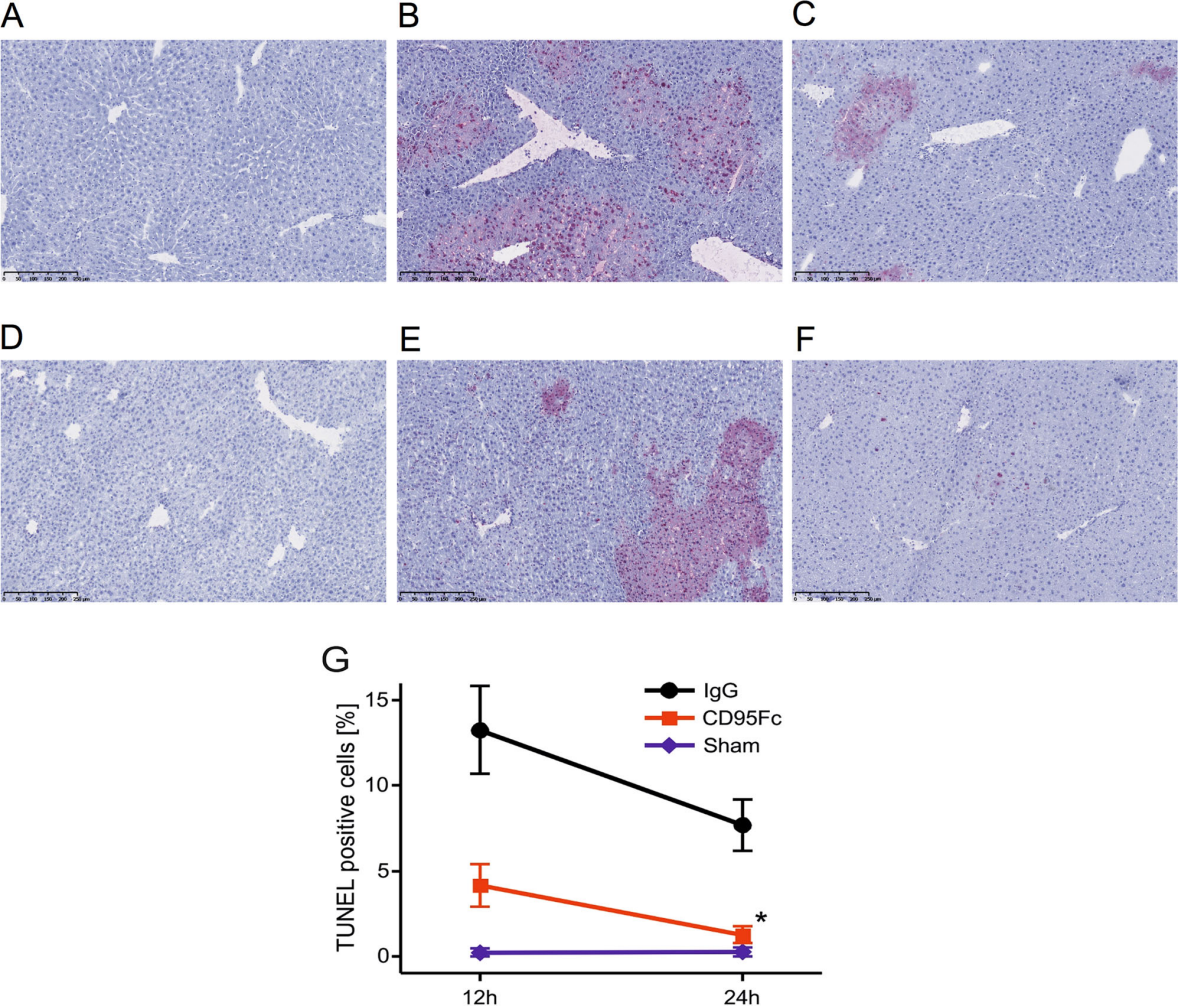
CD95L neutralization protects against apoptosis in ischemia-reperfusion injury of the liver Liver injury was induced by 45 min of warm ischemia of the median and left lateral liver lobe after prior application of two IV injections of CD95Fc (30 mg/kg) or the same volume of IgG (30 mg/kg) antibody at 12 h and at 30 min before ischemia. After 12 h and again after 24 h of reperfusion, liver tissue was harvested and processed for histopathology. Representative TUNEL-stained liver sections are presented (×100 magnification). **a** sham, reperfusion 12 h; **b** IgG, reperfusion 12 h; **c** CD95Fc, reperfusion 12 h; **d** sham, reperfusion 24 h; **e** IgG, reperfusion 24 h; **f** CD95Fc, reperfusion 24 h. **g** Percentage of TUNEL-positive tissue surface area [%] (mean ± S.D., *n* = 6. *p < 0.0001, MANOVA, between-subject-effect compared to IgG, time points 12 and 24 h were considered as repeated measurements)

Liver histopathology was assessed for signs of hepatic injury (Fig. 3a–f). CD95Fc was shown to significantly decrease the extent of hepatocellular necrosis from 25 ± 6% in the controls to 10 ± 4% (*p* = 0.0008) 12 h after reperfusion and also from 20 ± 4% in the controls to 8 ± 3% (*p* = 0.0002) 24 h after reperfusion (Fig. 3g). The distribution of necrosis around perivenous hepatocytes as a reflection of zonal oxygen gradients in both groups did not differ. To ascertain the extent of apoptosis, we performed TUNEL staining of the liver sections to determine DNA fragmentation (Fig. 4a–f). Mice, that were administered CD95Fc, showed a 3.25-fold decrease (*p* < 0.0001) in the number of TUNEL-positive cells compared to the control mice 12 h after reperfusion and an eight-fold decrease (*p* < 0.0001) in the number of TUNEL-positive cells 24 h after reperfusion (Fig. 4g).

In summary, pathological examination revealed better preserved lobular structure and significantly less necrosis and less apoptosis after CD95L neutralization in the treatment group in comparison to the control group. Thus, CD95L neutralization diminished otherwise abundant hepatocellular necrosis/apoptosis, manifested as reduced frequency of TUNEL-positive cells within the liver.

### CD95L inhibition using CD95Fc reduces caspase activity in liver IRI

**Fig. 5:**
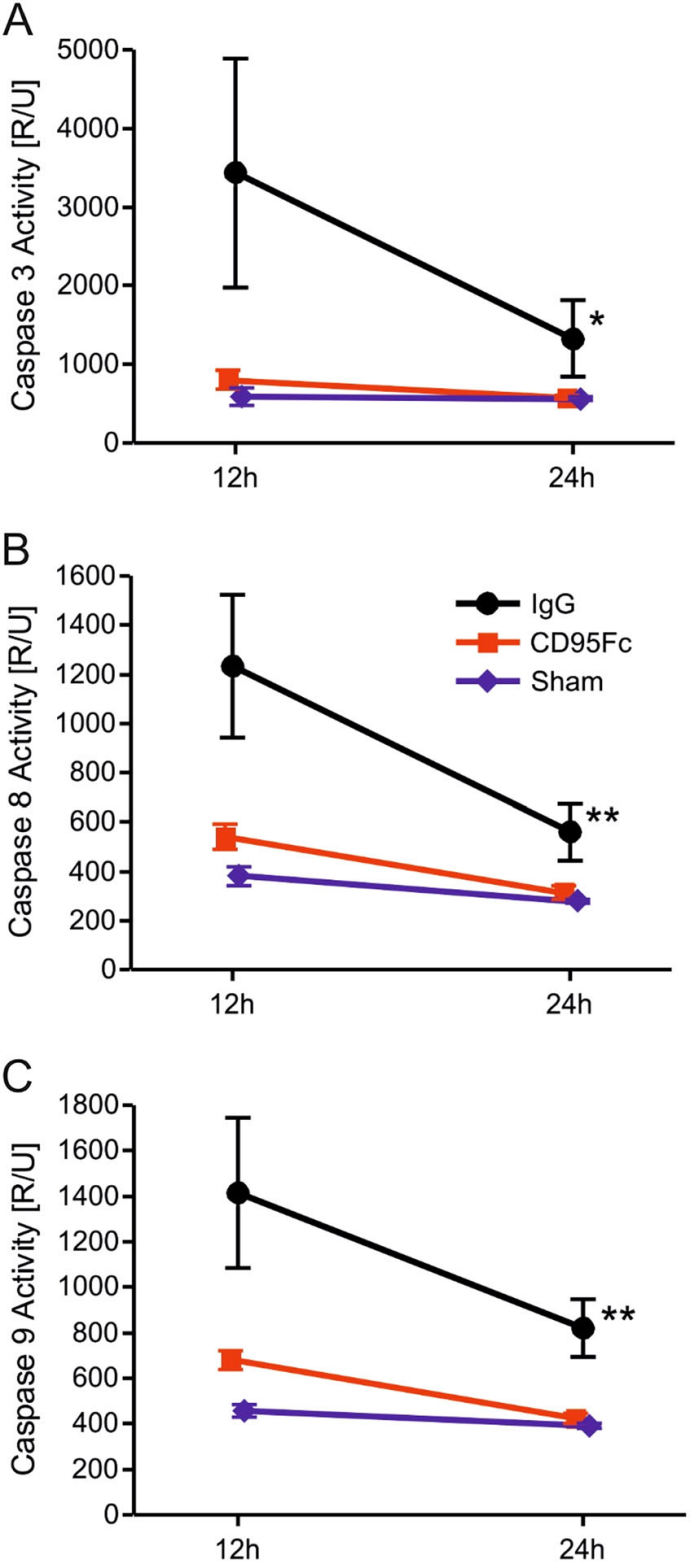
Caspase activity is reduced by application of CD95Fc After 45 min of warm ischemia of the median and left lateral liver lobe, liver tissue was collected after 12 h and again after 24 h of reperfusion from CD95Fc-treated and control mice and processed for further analysis. Caspase 3 activity **a**, caspase 8 activity **b** and caspase 9 activity **c** were assessed by fluorometric assays (mean ± S.D., *n* = 6. *p < 0.001, **p < 0.0001, MANOVA, between-subject-effect compared to IgG, time points 12 and 24 h were considered as repeated measurements)

The treatment of mice with CD95Fc significantly reduced the activity of caspase-3 by 4.3-fold (*p* = 0.0013), of caspase-8 by 2.3-fold (*p* = 0.0002), and of caspase-9 by 2.1-fold (*p* = 0.0003) in comparison to the levels in the control mice. These results were observed after 12 h of reperfusion in homogenates of post ischemic liver tissue. In line with a reduction of caspase activity after 12 h of reperfusion, we also observed a 2.3-fold (*p* = 0.0039), 1.8-fold (*p* = 0.0005) and 1.9-fold (*p* < 0.0001) decrease of caspase activity of caspase-3, caspase-8, and caspase-9 activity in CD95Fc-treated mice after 24 h of reperfusion (Fig. 5a–c).

In summary, neutralization of CD95L protects the liver against IRI, attenuates liver damage, diminishes apoptosis and necrosis and thus prevents acute liver failure in mice. These data highlight the functional importance of the CD95/CD95L death receptor ligand pathway in protecting the liver from IRI-induced injury.

## Discussion

Findings obtained in the present study allow us to propose a new model for the molecular mechanisms of action of the CD95L/receptor signaling pathway in IRI of the liver. Ischemia followed by reperfusion in the liver is a source of morbidity and mortality after liver transplantation, resection surgery, sepsis or hemorrhagic shock. IRI is a series of events that result in cell death by apoptosis and/or necrosis and serious dysfunction of hepatocytes^10,29^. Despite intensive studies, interventions with clinically proven efficacy remain to be developed. The understanding of the molecular mechanisms underlying cell death in hepatic IRI will provide the basis for the development of new therapeutic strategies for prevention of IRI and improvement of survival of the graft and patient.

Our manuscript describes the key role of the CD95L/receptor system in the mediation of cell death after IRI in the mouse liver. We show here that neutralization of CD95L with the CD95L-blocker, CD95Fc, is effective in protecting mice from acute liver failure due to IRI. CD95Fc could therefore emerge as a new pharmacological therapy in many clinical settings, such as liver resection, transplantation surgery, sepsis or shock.

The serum levels of ALT, AST and LDH are subject to rapid change after IRI to the liver and are clinically used as indicators of the severity of tissue/liver damage. Therefore, a distinct temporal pattern of enzyme levels due to enzyme-specific half-lives following IRI are crucial parameters in investigating and describing hepatic failure^30^. We observed an outstanding decrease of the levels of ALT, AST and LDH in CD95Fc-treated animals in comparison with IgG-treated control animals.

On the tissue level, the injury detected after transient clamping of hepatic blood flow, is determined by a complex network and cross talk of multiple molecular and cellular interactions. The result of these processes is an initial phase characterized by the release of ROS and proinflammatory mediators by both Kupffer and sinusoidal endothelial cells^6,31–33^. ROS lead to oxidative damage, induction of p53, apoptosis and necrosis of hepatocytes and endothelial cells. The late phase (6–48 h after reperfusion) is characterized by neutrophil-mediated inflammatory responses^23,31,32,34–40^. Thus, pathways regulating the cellular redox equilibrium, p53-dependent apoptosis and cellular death receptors represent potential targets for novel pharmaceutical interventions to protect hepatocytes fromIRI-induced cell death.

The death receptors known to mediate hepatocyte death include the CD95 (Apo-1/Fas), the tumor necrosis factor receptor 1 (TNF-R1), the tumor necrosis factor-related apoptosis-inducing ligand (TRAIL)-receptor 1 (TRAIL-R1), and the TRAIL-receptor 2 (TRAIL-R2), all of which are ubiquitously expressed in the liver^7,29,41–44^. Death receptors are activated by their corresponding ligands (CD95L, TNFα, and TRAIL), which subsequently trigger intracellular signaling pathways^7,8,45,46^.

ROS, which are produced by both the Kupffer and sinusoidal endothelial cells soon after reperfusion, are key players in inducing the up-regulation of CD95L in hepatocytes via the activation of nuclear factor-κB (NF-κB)^41,47^. Furthermore, CD95L is expressed on the cell surface of activated lymphocytes. Soluble forms of CD95L, released by polymorphonuclear cells (PMN), may also be present in the serum^15,17,18,48,49^.

The interaction of CD95 with its ligand CD95L plays a predominant role in apoptosis of the liver^50^. In a landmark study, the application of the agonistic anti-CD95 antibody Jo2, lead to massive apoptotic cell death of hepatocytes, resulting in imminent hepatic failure in mice^51,52^. In a study with a rat model of liver IRI, the number of apoptotic and CD95 positive hepatocytes gradually increased after reperfusion in parallel with an increase in the number of neighboring infiltrating CD95L positive cells. There was a massive intrusion of lymphocytes, monocytes, macrophages, PMN, and NK cells, predominantly around the central vein, 6 h after reperfusion. The infiltration of inflammatory cells peaked after 12 h with a concomitant rise in the quantity of the liver enzymes in the serum, thereby indicating hepatocellular damage^14^. The activation of the CD95/CD95L pathway, as well as the kinetics of the liver enzymes with a peak at 12 h, are in line with our results in a mouse non-lethal hepatic IR model. The prominent effects of therapeutical CD95 L neutralization in our study may thus be explained by effects on both—apoptotic cell death and inflammation. Therefore, the therapeutical neutralization of CD95L might be applicable for a more global clinical application beyond the transplant and surgical setting in ischemia/reperfusion conditions like sepsis and shock.

The results of our study provide further evidence that the CD95 signaling pathway and consecutive activation of caspases play a major role in the mechanism of hepatic IRI. In our experiments, we demonstrated that interference with this pathway via the inhibition of the CD95L with CD95Fc, thereby inhibiting activation of the CD95 receptor, leads to a dramatic reduction in the amount of liver injury as shown by amelioration of histology, inhibition of caspase activation, reduction of apoposis and necrosis and promotion of tissue regeneration.

Of utmost clinical importance is the fact that the antibody that we have applied is a new fully human fusion protein that consists of the CD95 receptor and the Fc domain of an IgG antibody. This antibody has been developed for the treatment of solid tumors and malignant hematological diseases and has been evaluated in the treatment of glioblastoma (phase II trial) and myelodysplastic syndromes (phase I trial). The excellent tolerability of this antibody was shown in a double-blind, placebo-controlled phase I trial in healthy volunteers. This makes the antibody a promising tool as a targeted therapy for IRI. Proof-of-concept trials will have to be set up to evaluate the efficacy of this compound in the treatment of patients with IRI.

In summary, our data highlight the functional importance of the CD95/CD95L signaling pathway in the mediation of IRI of the liver. Furthermore, and of clinical relevance, we provide evidence that neutralization of CD95L exerts potent hepatoprotective effects in vivo. These findings can be translated into therapeutic use for liver protection in many conditions, such as liver transplantation, partial hepatectomy, shock, sepsis and acute liver failure of other etiologies. Hence, neutralization of CD95L may serve as a new targeted therapy to attenuate liver ischemia reperfusion injury^53–59^.
